# SARS-CoV-2 pneumonia with large pneumothorax—a case report

**DOI:** 10.1186/s43168-022-00113-1

**Published:** 2022-01-29

**Authors:** Muhammad Hassan, Fibhaa Syed, Naveed Ullah Khan, Zakir Jan, Hafiza Faiza Mushtaq, Nadir Hussain, Mazhar Badshah

**Affiliations:** 1 Shaheed Zulfiqar Ali Bhutto Medical University (SZABMU), Islamabad, Pakistan; 2 Department of Neurology, SZABMU, Islamabad, Pakistan; 3 Department of Internal Medicine, SZABMU, Islamabad, Pakistan; 4 Department of Gynecology & Obstetrics, SZABMU, Islamabad, Pakistan; 5 Punjab Institute of Neurosciences (PINS), Lahore General Hospital, Lahore, Pakistan; 6 Department of Neurology, PINS, Lahore, Pakistan

**Keywords:** COVID-19, Pneumothorax, SARS-CoV-2

## Abstract

The pandemic of severe acute respiratory virus (SARS-CoV-2) is characterized by respiratory symptoms with serious consequences, mainly associated with pneumonia and extreme ARDS. There is a lack of data about pneumothorax associated with COVID-19 infections in current literature. Radiological features in SARS-CoV-2 include subpleural bilateral ground-glass appearance and many areas of irregular consolidation in the lungs. We here present a case of SARS-CoV-2 that was complicated by acute pneumothorax, and despite prompt treatment, that patient could not be saved. A 55-years-old male with no previous lung disease or any other history confirmed to have SARS-CoV-2 pneumonia developed a large pneumothorax on the third day of his presentation and was immediately intubated via a chest tube but could not be saved. SARS-CoV-2 by RT-PCR was positive. The patient expired around 12 h after chest intubation. Recommended treatment could not be started yet as the patient expired before it could be decided.

## Background

COVID pandemic hitting world’s health system through virulence and infectivity via aerosol transmission from December 2019 till date. Early detection and isolation of infected people, sterilization of the areas with disinfectants, and symptomatic treatment is the most successful way to combat the COVID outbreak [1]. Radiological evidence of pneumonia with clinical symptomatology plays a vital role in diagnosis [2]. Unilateral or bilateral ground-glass appearance with septation or reticulation is the most commonly found abnormality on high resolution computed tomography (HRCT) chest [2]. In addition, peripheral consolidation, inverted halo sign (atoll sign), air bubble sign, subpleural line, flagstone appearance, air bronchogram, and lymphadenopathy are among the HRCT findings [2]. In this study, we detected COVID-19 pneumonia with fever, cough and in isolation after chest pain and on repeat HRCT, a large left-sided pneumothorax was found.

## Case presentation

**Fig. 1:**
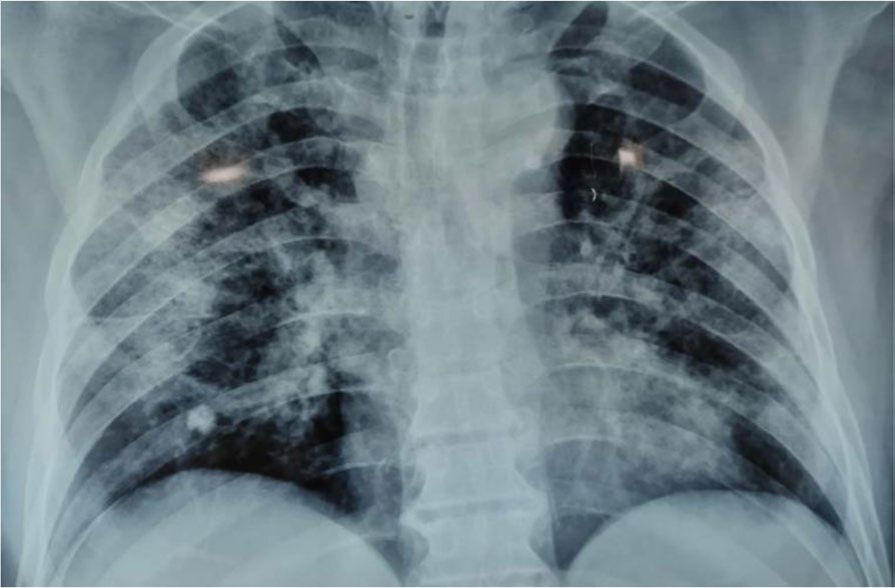
Initial chext X-ray at presentation to the ER which shows B/L diffuse patchy infiltrates

**Fig. 2:**
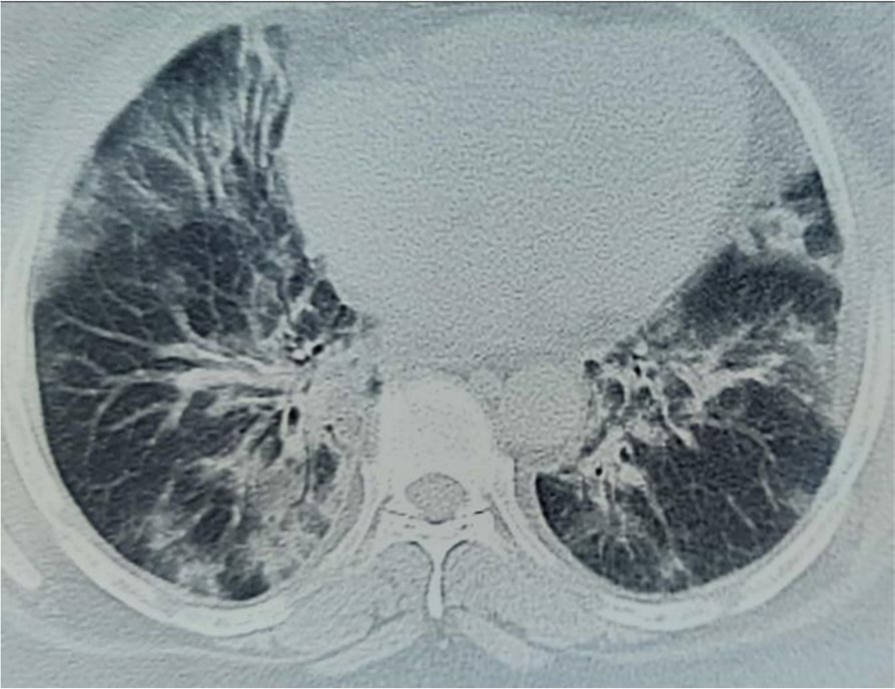
HRCT done on presentation to the ER that shows B/L ground glass infiltrates with patchy consolidations involving mainly the peripheries

**Fig. 3:**
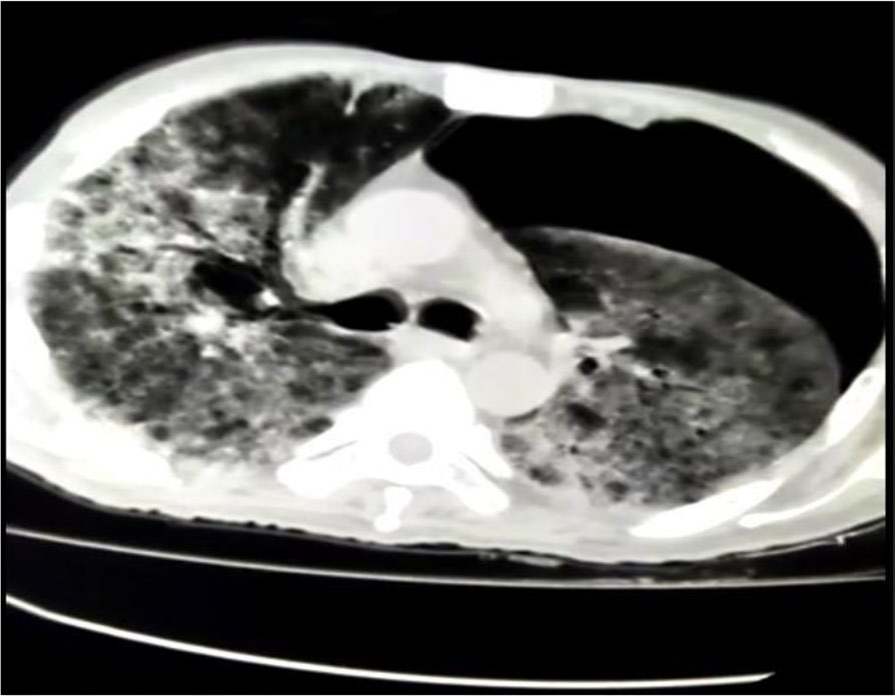
Large left-sided pneumothorax with typical COVID-19 lungs infiltrate

A 55-year, non-smoker patient with no previous known co-morbid presented to the emergency department with the complaint of fever, cough, and shortness of breath for the last four days. His general condition was good. He was conscious, cooperative, and orientated with a Glasgow Coma Scale (GCS) of 15/15. Blood pressure was 110/70 mmHg, pulse 92/min, respiratory rate 20/min, fever 38.5°C, oxygen saturation (SpO2) with finger probe was 85% at 20L of oxygen with dual oxygen support (15 L/min via face mask and 5 L/min via nasal prong) in the emergency room(ER). During his physical examination in the ER, his breathing sounds decreased on the left. Initial blood tests in the ER showed a WBC 13,150/μL, NEU 62%, lymphocyte 9.6/L, CRP 238 mg/L, serum ferritin 580 ng/mL, and serum D-Dimer 1940 ng/mL. The X-ray chest at presentation showed bilateral peripheral patchy consolidations and ground-glass opacities (Fig. 1). HRCT confirmed these findings, typical of COVID-19 pneumonia (Fig. 2). He was shifted to the isolation ward with oxygen support and started treatment as per the isolation protocol of COVID-19. Two days after his presentation, he developed severe chest pain on the left side. Repeat HRCT showed extensive ground-glass opacification with interlobular reticulations/septal thickening in bilateral lungs along with left-sided large pneumothorax, as shown in Fig. 3.

A pulmonology consult was done, and the patient had an urgent thoracostomy. Laboratory investigations were repeated that showed a marked increase in CRP and D-dimer levels, i.e., 528 mg/L and 5000 ng/mL, respectively. Blood culture and sensitivity report, sent from ER, came out negative. The patient’s polymerase chain reaction analysis of the nasopharyngeal swab for SARS-CoV-2 came out to be positive. The patient was planned for treatment with plasma therapy or Tocilizumab. He was given ceftriaxone 1gm intravenously (IV) twice daily (BD), azithromycin 500 mg intravenously once daily (OD), omeprazole 40 mg IV x OD, enoxaparin 40 mg subcutaneously OD, umifenovir 300 mg three times daily, and methylprednisolone 40 mg BD. The patient was not maintaining oxygen saturation on 30L oxygen via a non-rebreather mask with reservoir and nasal prongs followed by shifting to a non-invasive Bi-level ventilation support device. He expired after 12 h of management after thoracostomy before it could be decided if he may or may not be shifted to the Intensive care unit for ventilatory support. Ethical approval seeks from the patient’s attendant for publication.

## Discussion

Pneumothorax means the air in the pleural cavity, which in some instances may lead to a complete collapse of the lungs and can be fatal. Primary spontaneous pneumothorax usually occurs in patients with no prior lung disease, while secondary spontaneous pneumothorax occurs in a diseased lung. Whatever the cause, if not treated promptly, it may lead to decreased oxygenation and subsequent patient death due to hemodynamic instability. Our case is unique in the sense that there have been very few COVID-19 cases so far in the literature complicated by pneumothorax, with one case of spontaneous pneumomediastinum reported by a team of Chinese clinicians [3]. As our patient had no prior history of any lung and his radiological workup was also not suggestive of any prior lung damage, we assumed that the COVID-19 may have caused this severe fatal complication in our patient.

Our patient calls to attention the possibility of pneumothorax as a complication in a deteriorating COVID-19 patient with worsening or persistent hypoxia. Previously it has been thought that pneumothorax may be a wrong prognostic marker in COVID-19 patients [4, 5], as in our patient who, despite the management protocol for pneumothorax, was unable to survive and expired 12 h after a chest tube was passed.

Early bedside techniques and investigations may be crucial in such cases. As per the treatment protocol of many local and international hospitals, any suspected or confirmed COVID-19 patient who is deteriorating and not maintaining SpO2 on dual flow oxygen are either started on Bi-PAP or put on a ventilator, which in our case may have even worsened the patient’s condition faster. Physicians should be cautious, keeping in mind that such complications may be possible in COVID-19 infection, and avoid a purely algorithmic approach to critically unwell patients. Due to fear of the spread of the SARS-CoV-2 virus, most physicians are reluctant to properly examine the patients and auscultate their chest, which may lead to the initiation of wrong treatment strategies, which may have devastating effects on the patients.

In summary, while protocols remain critical in managing critically unwell patients, they cannot replace thorough history-taking and clinical examination.

## Conclusion

The treating position should open their minds to all kinds of complications in suspected or confirmed COVID-19 patients as a new disease. Pneumothorax should be investigated in acutely deteriorating COVID-19 patients with persistent hypoxemia.

In patients with suspected COVID-19, pneumothorax should be considered a cause of acute decompensation. Despite hospital protocols for managing COVID-19 patients, a physician should still follow proper steps of clinical history and examination in their assessment.

## Data Availability

Yes
